# A trade off between catalytic activity and protein stability determines the clinical manifestations of glucose-6-phosphate dehydrogenase (G6PD) deficiency

**DOI:** 10.1016/j.ijbiomac.2017.06.002

**Published:** 2017-11

**Authors:** Usa Boonyuen, Kamonwan Chamchoy, Thitiluck Swangsri, Thanyaphorn Junkree, Nicholas P.J. Day, Nicholas J. White, Mallika Imwong

**Affiliations:** a Department of Molecular Tropical Medicine and Genetics, Faculty of Tropical Medicine, Mahidol University, Bangkok, 10400, Thailand; b Mahidol-Oxford Tropical Medicine Research Unit, Faculty of Tropical Medicine, Mahidol University, Bangkok, 10400, Thailand; c Centre for Tropical Medicine, Nuffield Department of Medicine, University of Oxford, Oxford, United Kingdom

**Keywords:** G6PD deficiency, Thermal stability, Catalytic activity

## Abstract

Glucose-6-phosphate dehydrogenase (G6PD) deficiency is the most common polymorphism and enzymopathy in humans, affecting approximately 400 million people worldwide. It is responsible for various clinical manifestations, including favism, hemolytic anemia, chronic non-spherocytic hemolytic anemia, spontaneous abortion, and neonatal hyperbilirubinemia. Understanding the molecular mechanisms underlying the severity of G6PD deficiency is of great importance but that of many G6PD variants are still unknown. In this study, we report the construction, expression, purification, and biochemical characterization in terms of kinetic properties and stability of five clinical G6PD variants—G6PD Bangkok, G6PD Bangkok noi, G6PD Songklanagarind, G6PD Canton + Bangkok noi, and G6PD Union + Viangchan. G6PD Bangkok and G6PD Canton + Bangkok noi showed a complete loss of catalytic activity and moderate reduction in thermal stability when compared with the native G6PD. G6PD Bangkok noi and G6PD Union + Viangchan showed a significant reduction in catalytic efficiency, whereas G6PD Songklanagarind showed a catalytic activity comparable to the wild-type enzyme. The Union + Viangchan mutation showed a remarkable effect on the global stability of the enzyme. In addition, our results indicate that the location of mutations in G6PD variants affects their catalytic activity, stability, and structure. Hence, our results provide a molecular explanation for clinical manifestations observed in individuals with G6PD deficiency.

## 1 Introduction

Glucose-6-phosphate dehydrogenase (G6PD, E.C. 1.1.1.49) is a metabolic enzyme that catalyzes the first and rate-limiting step of the pentose phosphate pathway, converting d-glucose-6-phosphate (G6P) to 6-phosphoglucono-δ-lactone and producing the reduced form of nicotinamide adenine dinucleotide phosphate (NADPH) [1], which plays a critical role in protecting cells against oxidative stress by regulating the levels of reduced glutathione [2]. G6PD is particularly important in erythrocytes because it is the sole production source of reducing equivalents. G6PD deficiency is a hereditary genetic defect that is the most prevalent polymorphism and enzymopathy in humans, affecting more than 400 million people worldwide [3]. A wide spectrum of clinical manifestations of G6PD deficiency is observed, which includes favism, hemolytic anemia, chronic non-spherocytic hemolytic anemia (CNSHA), spontaneous abortion, and neonatal hyperbilirubinemia resulting in neonatal kernicterus, which can be fatal [4], [5], [6].

G6PD variants have been classified into five different classes according to their residual enzyme activity, ranging from Class I G6PD variants, which show less than 5% residual activity and the most severe clinical phenotypes, to Class V G6PD variants, which show increased enzyme activity but no clinical manifestations [7]. Currently, 217 G6PD deficiencies have been identified at the DNA level, and over 400 variants have been described on the basis of their biochemical properties. Most of the variants reported are single-point mutations causing single amino acid substitution. Double and triple mutants have also been reported, but with much lower frequencies [8], [9]. Unfortunately, only about 10% of G6PD variants have been structurally and functionally characterized.

Malaria drugs such as primaquine, sulfanilamide, and sulfadoxine have been reported to cause hemolysis in G6PD-deficient individuals [10]. Therefore, the hemolytic toxicity of G6PD variants has an impact on malaria treatment. In particular, it has raised a significant concern regarding malaria elimination because of the widespread prevalence of G6PD deficiency in regions such as Southeast Asia and Africa, where malaria is endemic [11], [12], [13]. The clinical hemolytic episode of G6PD deficiency is varied, ranging from mild and self-limiting to severe and even fatal, depending on exposure and the G6PD genotype. A cohort study reported the effect of primaquine treatment on female patients with malaria carrying G6PD Mahidol, a mild G6PD variant that is common in Southeast Asia [14]. The study found that a high daily dose of primaquine has the potential to cause clinically significant hemolysis in women with heterozygous G6PD who show the normal G6PD phenotype by point-of-care test. The association between G6PD deficiency and the hemolytic phenotype indicates reduced NADPH production, which could be caused by unstable or catalytically inactive G6PD. Damaged or unstable G6PD molecules cannot be substituted because erythrocytes are non-nucleated cells. Furthermore, erythrocytes, as oxygen carriers, are highly vulnerable to oxidative stress if they are catalytically deficient. This suggests that more information on the hemolytic risk to individuals with G6PD deficiency and different G6PD activities is required. To date, the relation between G6PD genotype and enzyme activity has not been established. Indeed, such information is crucial to ensure the safety of treatment for malaria patients with G6PD deficiency.

In Southeast Asia, where malaria is endemic, the prevalence of G6PD deficiency ranges between 3% and 18% depending on the area [11], [12], [15], [16], [17], [18], [19], [20], [21], [22]. Several G6PD variants have been described, including G6PD Mahidol, G6PD Viangchan, G6PD Union, G6PD Canton, G6PD Songklanagarind, G6PD Bangkok, G6PD Bangkok noi, G6PD Mahidol + Viangchan, G6PD Canton + Bangkok noi, and G6PD Union + Viangchan [12], [15], [19], [20]. Almost all of these G6PD variants are Class II or Class III variants. Class I variants are rare and comprise only two—G6PD Bangkok and G6PD Canton + Bangkok noi. Both variants are reported to be associated with CNSHA [23], [24]. Because G6PD Canton is a Class II variant and therefore cannot contribute to severe hemolysis on its own in individuals with the G6PD Canton + Bangkok noi variant, the role of G6PD Bangkok noi should be considered, which remains to be elucidated [22]. Previously, we have functionally and biochemically characterized G6PD Mahidol, G6PD Viangchan, and G6PD Mahidol + Viangchan [25]. We found that the catalytic efficiency and protein instability contribute to the clinical phenotypes of G6PD variants. In the present study, we intended to extend our understanding of the molecular mechanisms underlying the observed clinical manifestations to other G6PD variants found in Thailand and other countries in Southeast Asia. We selected five clinical G6PD variants—G6PD Bangkok, G6PD Bangkok noi, G6PD Songklanagarind, G6PD Canton + Bangkok noi, and G6PD Union + Viangchan—and elucidated their construction, overexpression, purification, and functional characterization. In addition, two other G6PD variants (G6PD Union and G6PD Canton) were also characterized in order to investigate the cooperation of the two mutations in producing the observed clinical phenotypes.

## 2 Materials and methods

### 2.1 Construction of recombinant G6PD variants by site-directed mutagenesis

Seven clinical variants—G6PD Bangkok (nt G825C, Lys275Asn), G6PD Bangkok noi (nt T1502G, Phe501Cys), G6PD Songklanagarind (nt T196A, Phe66Ile), G6PD Union (nt C1360T, Arg454Cys), G6PD Canton (nt C1376T, Arg459Leu), G6PD Canton + Bangkok noi (nt C1376T + T1502G, Arg459Leu + Phe501Cys), and G6PD Union + Viangchan (nt C1360T + G781A, Arg454cys + Val291Met)—were generated using site-directed mutagenesis, with a pET28-G6PD wild-type plasmid being used as a template. The polymerase chain reaction (PCR) mixture (50 μL) comprised 1 × KAPA HiFi reaction buffer, 50 ng of template plasmid, 100 ng of forward and reverse primers, 0.3 μM of each dNTP mix, and 1 U of KAPA HiFi DNA polymerase (Kapa Biosystems); the primers used to generate the G6PD variants are listed in Table 1. The cycling parameters for site-directed mutagenesis are as follows: 1 cycle of 95 °C for 5 min and 16 cycles of 98 °C for 20 s, 55 °C for 15 s, and 68 °C for 3 min and 30 s. The PCR products of site-directed mutagenesis went through Dpn I digestion at 37 °C for 2 h to digest the parental DNA. Thereafter, the DNA was transformed into DH5α cells, which were plated on LB agar plates containing 50 μg/mL of kanamycin. All constructs were verified by bidirectional DNA sequencing and restriction digestion to confirm that the desired recombinant plasmids were obtained.

**Table 1:** List of primers used in site-directed mutagenesis.

Primer	Sequence
G6PD Bangkok Forward	5′ gccatggaga accccgcctc cacc 3′
G6PD Bangkok Reverse	5′ ggtggaggcg gggttctcca tggc 3′
G6PD Bangkok noi Forward	5′ agtgggttgc cagtatgagg gcac 3′
G6PD Bangkok noi Reverse	5′ gtgccctcat actggcaacc cact 3′
G6PD Songklanagarind Forward	5′ cccgaaaaca ccatcatcgt gggc 3′
G6PD Songklanagarind Reverse	5′ gcccacgatg atggtgtttt cggg 3′
G6PD Union Forward	5′ ccagatgcac ttcgtgtgca gcga 3′
G6PD Union Reverse	5′ tcgctgcaca cgaagtgcat ctgg 3′
G6PD Viangchan Forward	5′ gatgagaagg tcaagatgttgaaatgcatc 3′
G6PD Viangchan Reverse	5′ gatgcatttc aacatcttga ccttctcatc 3′
G6PD Canton Forward	5′ gacgagctcc ttgaggcctg g 3′
G6PD Canton Reverse	5′ ccaggcctca aggagctcgt c 3′

### 2.2 Expression and purification of the G6PD variants

Expression of the G6PD variants was performed as described previously [25]. A single colony of BL21 (DE3) carrying the desired plasmid was inoculated in 5 mL of LB medium in the presence of 50 μg/mL of kanamycin and was cultured overnight at 37 °C with 250 rpm of shaking. The overnight cultures were then inoculated into 1 L of fresh LB medium containing 50 μg/mL of kanamycin at a dilution of 1:100 and were grown at 37 °C with shaking at 250 rpm until the absorbance at 600 nm (OD_600_) reached 0.6–0.8. A final concentration of 1 mM of isopropyl β-d-thiogalactoside (IPTG, Merck) was used to induce G6PD expression. Bacterial cultures were further incubated at 20 °C with shaking at 180 rpm for 20 h before being harvested by centrifugation at 1000 × *g* for 15 min.

The purification of recombinant human G6PD variants was accomplished as previously described, with some modifications [25]. Cell pellets were resuspended in lysis buffer (20 mM sodium phosphate (pH 7.4), 300 mM NaCl, and 10 mM imidazole), broken up by sonication, centrifuged at 20,000 × *g* for 60 min at 4 °C in order to remove the cell debris, and the supernatant was collected. Thereafter, the supernatant was equilibrated with TALON Metal Affinity Resin (BD Biosciences) at 4 °C for at least 1 h. The unbound proteins were removed by washing with 20 mL of wash buffer (20 mM sodium phosphate (pH 7.4), 300 mM NaCl, and 20 mM imidazole). The elution of G6PD was performed using elution buffer (20 mM sodium phosphate (pH 7.4), 300 mM NaCl, and 40–400 mM imidazole). Imidazole was removed from the sample by overnight dialysis with 20 mM Tris-HCl (pH 7.5) containing 10% glycerol by using SnakeSkin Dialysis Tubing with a molecular weight cut-off of 10 kDa (Thermo Scientific). The purity of the recombinant protein was analyzed with 12% SDS-PAGE stained with Coomassie Brilliant Blue (Sigma-Aldrich). Protein concentration was determined using the Bradford assay [26]. The purified enzyme was stored in the presence of 10 μM NADP^+^ at −20 °C.

### 2.3 Determination of steady-state kinetic parameters

G6PD activity was assayed spectrophotometrically by monitoring the reduction of NADP^+^ at 340 nm by using a UV-2700 UV–VIS spectrophotometer (Shimadzu) as previously described [25]. The standard reaction mixture contained 20 mM Tris-HCl (pH 8.0), 0.01 M MgCl_2_, 200 μM NADP^+^, and 500 μM G6P. The reaction was initiated with the addition of the enzyme. Steady-state kinetic parameters were obtained by varying the concentration of one substrate (1–500 μM for NADP^+^ and 5–500 μM for G6P) and fixing the concentration of the second substrate at saturating concentration (200 μM for NADP^+^ and 500 μM for G6P). The experiment was performed in triplicate. The initial velocity obtained from the spectrophotometer was used to calculate the rate of product formation and was expressed as micromole of NADPH produced per minute per milligram of protein (μmole/min/mg), as calculated using the extinction coefficient of NADPH at 340 nm (6220 M^−1^ cm^−1^). Steady-state kinetic parameters, *K*_M_*, k*_cat_, and *V*_max_, were determined by fitting the data to the Michaelis–Menten equation by using GraphPad Prism (GraphPad Software).

### 2.4 Secondary structure analysis by circular dichroism (CD)

The secondary structure of the G6PD variants was evaluated spectroscopically by CD as previously described [25]. Far UV-CD spectra of the G6PD variants at a protein concentration of 0.35 mg/mL (in the absence of NADP^+^) were recorded using a Jasco spectrometer, model J-815, equipped with a Peltier temperature controller system in a 1 mm path-length quartz cuvette. The experiments were performed in triplicate at 25 °C, and the values were then averaged for each protein sample. The spectra were collected over a wavelength range of 190–260 nm at a scan rate of 50 nm/min.

### 2.5 Intrinsic fluorescence and 8-anilinonaphthalene-1-sulfonate (ANS)-binding analysis

After the removal of bound NADP^+^ by buffer exchanged with 20 mM Tris-HCl (pH 7.5) by using Amicon Ultra centrifugal filter devices (Millipore), the intrinsic fluorescence emission spectrum of the G6PD proteins was collected using a Synergy H1 hybrid reader (Biotek) with a 96-well plate at 25 °C. The excitation wavelength was 295 nm, and the emission spectra were recorded in the range of 300–400 nm.

The ability of G6PD proteins to bind to ANS was assessed after the removal of bound NADP^+^ as aforementioned. A 100 μM ANS was used for sample preparation and the concentration of G6PD was adjusted to 0.1 mg/mL. The G6PD proteins were incubated with ANS at 25 °C for 1 h before monitoring the emission spectra between 400 and 600 nm using an excitation wavelength of 395 nm in a Synergy H1 hybrid reader (Biotek). The experiment was performed in triplicate.

### 2.6 Thermal stability analysis

The thermal stability and unfolding of the G6PD variants was determined by following the change in the CD signal at 222 nm while varying the temperature from 20 °C to 80 °C at a scan rate of 1 °C/min. Bound NADP^+^ was removed from the purified enzyme and the concentration of G6PD was adjusted to 0.35 mg/mL. The experiment was performed in triplicate. The melting temperature (*T*_m_) was defined as the temperature at which half of the secondary structure unfolded and was calculated for each G6PD variant.

### 2.7 Thermal inactivation analysis

Thermal inactivation analysis was performed as previously described [25]. Removal of the bound NADP^+^ was accomplished for the purified enzyme by buffer exchanged as previously mentioned, and the enzyme concentration was adjusted to 0.2 mg/mL. The G6PD variants were incubated with varying concentrations of NADP^+^ (0, 1, 10, 100, and 1000 μM) at different temperatures ranging from 25 °C to 65 °C for 20 min and were then cooled down to 4 °C in a Thermocycler (Eppendorf). Residual activity of the enzyme was determined and was expressed as a percentage of the activity of the same enzyme incubated at 25 °C. The experiment was performed in triplicate.

### 2.8 Analysis of the stability of G6PD variants in the presence of guanidine hydrochloride (Gdn-HCl)

The stability of G6PD variants in the presence and absence of Gdn-HCl was determined. Bound NADP^+^ was removed as mentioned earlier, and the concentration of the enzyme was adjusted to 0.2 mg/mL. All samples were incubated in the presence of varying concentrations of Gdn-HCl (0.05, 0.1, 0.15, 0.2, 0.25, 0.3, 0.4, and 0.5 mM) at 37 °C for 2 h. Thereafter, the residual activity of the enzyme was measured and expressed as a percentage of the activity of the same enzyme incubated at 25 °C in the absence of Gdn-HCl. The experiment was performed in triplicate.

### 2.9 Trypsin digestion

The susceptibility of G6PD to trypsin was determined in the presence of different concentrations of NADP^+^ (1, 10, and 100 μM). Firstly, bound NADP^+^ was removed from the G6PD enzymes as mentioned earlier, and the protein concentration was adjusted to a concentration of 0.2 mg/mL. Trypsin (0.5 mg/mL) was added to the enzyme samples, which were incubated at 25 °C. The residual enzyme activity was examined at time intervals (5–150 min) and was expressed as a percentage of the activity of the same enzyme without incubation. The experiment was performed in triplicate.

## 3 Results and discussion

### 3.1 Steady-state kinetics of G6PD variants

**Fig. 1:**
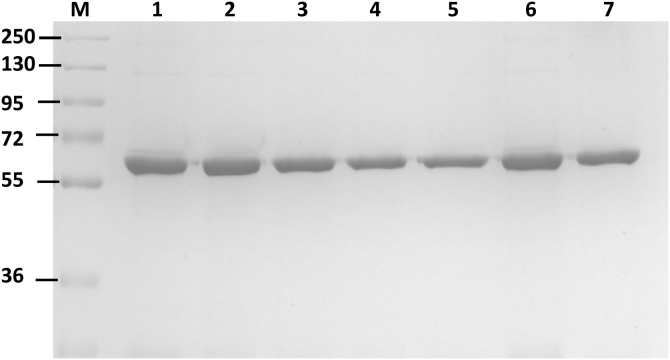
SDS-PAGE analysis of purifed recombinant G6PD variants: lane M, molecular mass marker protein; lane 1, G6PD Bangkok; lane 2, G6PD Bangkok noi; lane 3, G6PD Union; lane 4, G6PD Sonklanagarind; lane 5, G6PD Union + Viangchan; lane 6, G6PD Canton and lane 7, G6PD Canton + Bangkok noi.

**Fig. 2:**
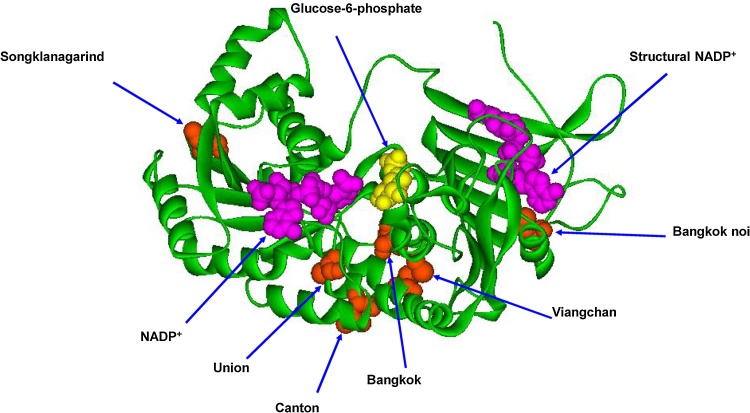
Three-dimensional structure of the human G6PD enzyme [29], [30]. The monomer is shown with NADP^+^ binding at structural and coenzyme binding sites (pink) and G6P site (yellow). The mutants are shown in CPK representation and labeled. The graphical representation was constructed using Discovery Studio Visualizer-Accelrys.

**Fig. 3:**
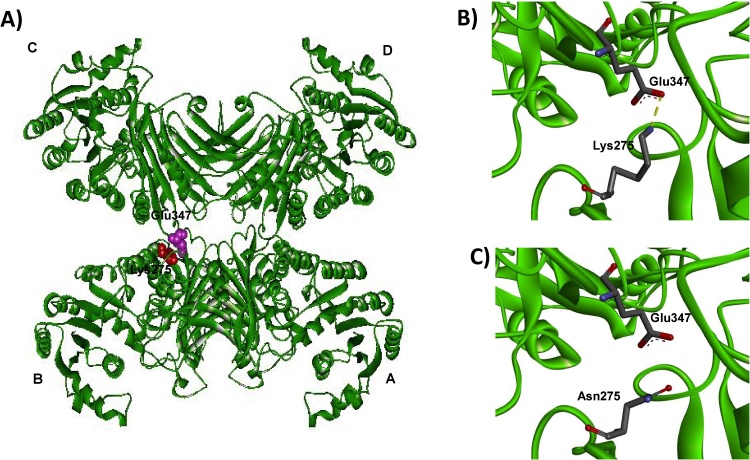
Three-dimensional structure of G6PD tetramer [29]. A) Tetrameric structure of G6PD wild-type. The four subunits are shown in green and residues forming salt bridge (Lys275 and Glu347) are shown in CPK representation. B) The salt bridge that links between subunit B and C of tetrameric structure in wild-type G6PD. C) The salt bridge is disrupted in G6PD Bangkok (Lys275Asn).

The clinical manifestations of G6PD deficiency could be caused by a reduction in catalytic efficiency or a reduction in the number of active enzyme molecules. To elucidate thoroughly the molecular mechanisms underlying the clinical phenotypes of G6PD variants, the seven recombinant human G6PD variants were successfully generated using site-directed mutagenesis and were expressed and purified to homogeneity by affinity chromatography (Cobalt resin); imidazole was removed by dialysis. The SDS-PAGE analysis of recombinant G6PD variants is shown in Fig. 1; the purity of purified enzymes is greater than 95%. Because a previous study has shown that His-tagging G6PD proteins does not affect their catalytic activity and stability, all G6PD variants were His-tagged in this study and were characterized without removing the tag [27]. To evaluate the effect of mutations on catalytic efficiency, steady-state kinetic parameters were determined for all of the seven clinical G6PD variants (Table 2). The two natural variants, G6PD Bangkok and G6PD Canton + Bangkok noi, showed no detectable enzymatic activity. Indeed, these two variants are classified as Class I variants exhibiting severe hemolysis with less than 5% residual activity. This study’s results are in good agreement with the clinical manifestations of CNSHA observed in patients carrying the G6PD Bangkok and G6PD Canton + Bangkok noi variants. These two G6PD variants also showed no detectable G6PD activity in patients [23], [24]. In contrast, it has been reported that a total loss of G6PD activity is embryonically lethal in mice [28]. G6PD Bangkok contains a mutation at residue 275, which is a part of the tetramer interface, substituting Lys with Asn (Fig. 2, Fig. 3) [29], [30]. In fact, Lys 275 forms a salt bridge with Glu 347, connecting the two dimers together. Substitution of a positively charged residue (Lys) with neutrally charged one (Asn) disrupts the salt bridge formation and may contribute to a complete loss of activity observed in the G6PD Bangkok variant.

**Table 2:** Steady-state kinetic parameters of recombinant human G6PD enzymes.

Construct	Class	Amino acid substitution	3D position	*k_cat_* (s^−1^)	*K_M_G6P* (μM)	*K_M_NADP^+^* (μM)	*k_cat_*/*K_M_G6P* (μM^−1^ s^−1^)	*k_cat_*/*K_M_NADP^+^* (μM^−1^ s^−1^)
Wild type	–	–	–	249 ± 9	47.8 ± 4.2	7.2 ± 1.8	5.2 ± 0.7	34.3 ± 2.9
Bangkok	I	Lys275Asn	αi-αj loop 273–287	ND	ND	ND	ND	ND
Bangkok noi	I	Phe501Cys	post αo	5.2 ± 0.3	71.2 ± 6.5	3.8 ± 0.4	0.07 ± 0.002	1.4 ± 0.1
Songklanagarind	II	Phe66Ile	βB 65–71	232 ± 7	45.3 ± 3.7	14.8 ± 2.1	5.1 ± 0.3	15.7 ± 1.2
Union	II	Arg454Cys	αm-αn loop 447–454	139 ± 3	10.5 ± 1.8	8.6 ± 1.2	13.2 ± 0.8	16.1 ± 1.1
Canton	II	Arg459Leu	αn loop 455–473	123 ± 4	23.4 ± 2.8	14.7 ± 1.1	5.6 ± 0.9	8.4 ± 0.7
Union + Viangchan	II/III	Arg454Cys + Val291Met	–a	109 ± 4	7.9 ± 0.7	6.5 ± 0.8	13.8 ± 1.5	16.8 ± 1.6
Canton + Bangkok noi	I	Arg459Leu + Phe501Cys	–a	ND	ND	ND	ND	ND

ND: no detectable enzyme activity.

a See individual 3D position.

The kinetic parameters of G6PD Canton ascertained in this study are in concordance with those published previously, with *k*_cat_, *K*_M_G6P, and K_M_NADP^+^ values of 123 ± 4 s^−1^, 23.4 ± 2.8 μM, and 14.7 ± 1.1 μM, respectively [22]. Because G6PD Canton is a Class II variant with a reduced catalytic efficiency of approximately 50% compared with the wild-type enzyme, it cannot solely contribute to the severe hemolysis observed for a double mutant such as G6PD Canton + Bangkok noi. Therefore, we characterized the other mutant, G6PD Bangkok noi, to elucidate the cooperative effect of Canton and Bangkok noi mutations for causing CNSHA in individuals carrying the G6PD Canton + Bangkok noi variant. G6PD Bangkok noi has a mutation at residue 501, which substitutes Phe with Cys. It is noteworthy to mention that G6PD Bangkok noi displayed a significant reduction in catalytic activity, with a *k*_cat_ value of 5.2 ± 0.3 s^−1^, which is approximately 50-fold lower than that of the wild-type protein. The binding affinity of substrates is also affected by the Bangkok noi mutation, with *K*_M_G6P and K_M_NADP^+^ values of 71.2 ± 6.5 μM and 3.8 ± 0.4 μM, respectively (Table 2). The influence of mutation on the clinical phenotypes of G6PD Bangkok noi is attributable to the alteration in the size of residue 501, which is located at the NADP^+^-binding pocket (Fig. 2). Thus, a combination of the effects of both G6PD Canton and G6PD Bangkok noi mutations leads to the CNSHA observed in individuals carrying the G6PD Canton + Bangkok noi variant.

The catalytic activity *k_cat_* of G6PD Songklanagarind, a Class II variant, was comparable to the native enzyme and had a value of 232 ± 7 s^−1^. Additionally, the affinity values of G6PD Songklanagarind and wild type enzyme for both substrates are in the same range [25], [31]. G6PD Songklanagarind contains amino acid alteration at residue 66, substituting Phe with Ile. Because residue 66 is not near the substrate- and structural NADP^+^-binding sites (Fig. 2), the catalytic efficiency of G6PD Songklanagarind is only slightly affected by this mutation.

For G6PD Union, its binding affinity to the two physiological substrates ascertained in this study was similar to that reported previously [31], [32]. In contrast, the catalytic activity of recombinant human G6PD Union determined in this study (*k_cat_* = 139 ± 3 s^−1^) was higher than that reported previously (*k_cat_* = 28.6 ± 1.3 s^−1^) [31], [32]. However, it is worth noting that the Class II variants, such as G6PD Viangchan, G6PD Mahidol + Viangchan, G6PD Vanua Lava, and G6PD Canton, show a reduced catalytic activity of around 40%–50% compared with the native enzyme, which is similar to the value obtained in the present study for G6PD Union [22], [25], [33]. Although the catalytic activity of G6PD Union determined in this study was different from that reported previously, the higher catalytic activity observed in this study could explain the distinction in the clinical severity of Arg454 mutation observed in G6PD Union and G6PD Andalus. G6PD Union (Arg454Cys) is a Class II variant, whereas G6PD Andalus (Arg454His) is a Class I variant with severe clinical phenotypes [21], [32], [34]. The *k_cat_* value of 139 ± 3 s^−1^ for G6PD Union is well fitted to milder hemolytic toxicity.

The double mutant and Class II variant G6PD Union + Viangchan showed a reduced catalytic activity of 43% compared with the wild-type enzyme. The decline in catalytic activity of this variant is also in the same range as that observed for other Class II while the binding affinity of G6PD Union + Viangchan to NADP^+^ was similar to that of the native enzyme (K_M_NADP^+^ = 6.5 ± 0.8 μM), its *K*_M_ for the G6P substrate was six-fold lower (*K*_M_G6P = 7.9 ± 0.7 μM) than that of the wild-type enzyme [25], [31], [35]. The catalytic activity of G6PD Union + Viangchan was higher than that of G6PD Mahidol + Viangchan reported previously, which is in agreement with the phenotypic characterization of these variants in G6PD-deficient individuals [15], [25].

Our results showed that the catalytic efficiency of the five natural G6PD variants is differentially affected by the location of the mutations. The effect of mutation on enzyme activity seems to be more prominent when the mutation is close to the dimer/tetramer interface (e.g., G6PD Bangkok) or is in proximity to the substrate- and structural NADP^+^-binding sites (e.g., G6PD Bangkok noi). Several studies have also demonstrated the differential impact of mutation sites on the activity of G6PD variants. For instance, the Pro172Ser mutation has a marked effect on the catalytic efficiency of G6PD Volendam, a Class I variant that produces CNSHA [36]. A mutation at residue 172 alters the binding affinity of both substrates as well as the catalytic activity of G6PD Volendam. The crystal structure of human G6PD indicates that the conformational position of Pro172 plays an important role in coenzyme binding because Pro172 is located at the conserved active site cleft of the enzyme [29]. Moreover, a recent study has reported that the catalytic activity is significantly reduced owing to the mutation at residue 257 in G6PD Zacatecas, which is also a Class I variant. The change of amino acid at position 257 from Arg to Leu dramatically affects the catalytic efficiency as it is located close to the G6P-binding site, causing a reduction of 70% in the catalytic activity compared with the wild-type enzyme [33]. However, a milder effect of mutation was observed in G6PD variants such as G6PD Canton, G6PD Viangchan, and G6PD Mahidol, wherein the mutations are neither part of the substrate- and structural NADP^+^-binding sites nor close to the dimer/tetramer interface [25], [33], [35]. However, the mutations that are located far from the substrate- and structural NADP^+^-binding sites and are not part of the dimer/tetramer interface could also contribute to severe clinical phenotypes by different mechanisms such as the defective in protein structure or stability.

### 3.2 Structural analysis of G6PD variants by circular dichroism (CD) and intrinsic fluorescence

**Fig. 4:**
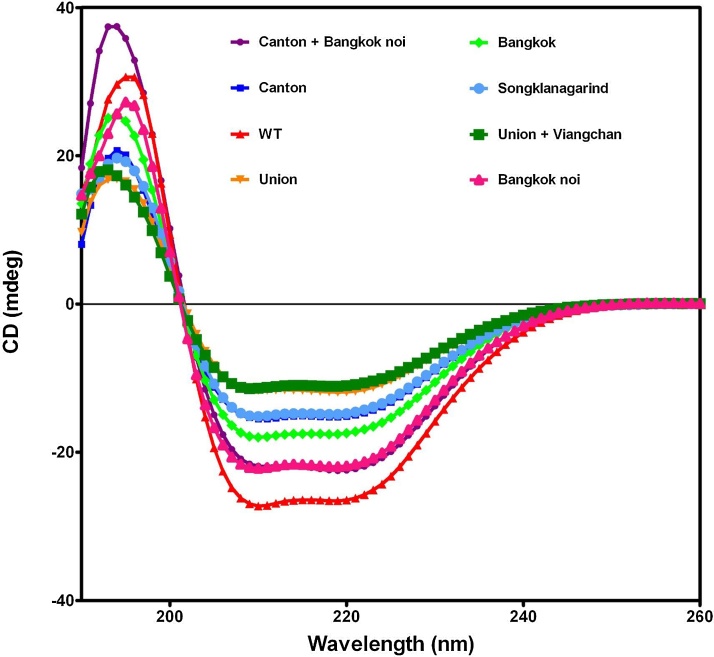
CD spectra in far-UV region of recombinant human G6PD variants.

In addition to the decrease in catalytic activity, protein instability also contributes to the hemolytic phenotype observed in G6PD-deficient individuals. Therefore, mutation-induced impairment of protein stability was also investigated. The effect of mutation on protein conformation was assessed by CD analysis. The secondary structure of wild-type G6PD and seven clinical G6PD variants was determined using the far-UV region (190–260 nm) and they all shared a similar pattern of CD spectra (Fig. 4), showing maximum absorption peaks at 208 and 222 nm, which is a characteristic of the α + β structure of the G6PD protein [29]. It is worth mentioning that wild-type G6PD showed the greatest negative peak at 222 nm while other G6PD variants showed less negative peak at 222 nm. This indicates that the mutation in each variant alters the secondary structure of the protein. Interesting, it was observed that G6PD Union and G6PD Union + Viangchan showed the least negative peak at 222 nm, suggesting that mutation at residues 454 (Union) caused a significant change in the secondary structure of the protein. Alteration in secondary structure observed in the double mutant is unlikely to be caused by G6PD Viangchan because this mutation has no effect on the secondary structure of the protein [25].

**Fig. 5:**
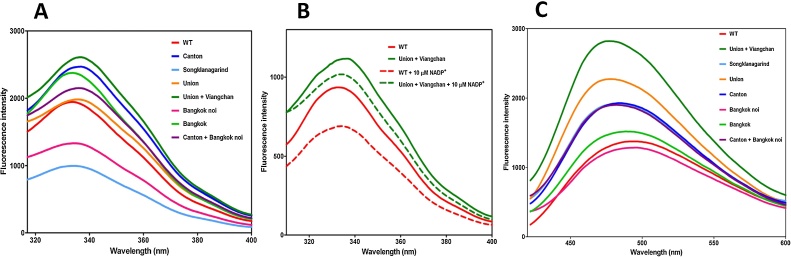
Fluorescence emission spectra of the G6PD variants. (A) Intrinsic fluorescence spectra of recombinant human G6PD wild type and seven clinical variants in the absence of NADP^+^. (B) Representative intrinsic fluorescence spectra of recombinant human G6PD wild type and G6PD Union + Viangchan in the absence (solid line) and presence of 10 μM NADP^+^. (C) ANS fluorescence spectra of G6PD variants in the absence of NADP^+^.

Considering that G6PD contains seven residues of tryptophan per monomer, we assessed structural alterations by monitoring changes in the intrinsic fluorescence emission of the tryptophan residues. Therefore, the intrinsic fluorescence and the ability to bind to 8-anilinonaphthalene-1-sulfonate (ANS) of the G6PD variants were determined (Fig. 5). At an excitation wavelength of 295 nm, the fluorescence emission mainly belonged to tryptophan over tyrosine and phenylalanine. Because tryptophan is rarely present in proteins, analyzing the intrinsic fluorescence of this amino acid is a sensitive method to measure the conformational state of the residue. Moreover, changes in intrinsic fluorescence emission have been widely used to assess conformational changes in proteins [37], [38]. The intrinsic fluorescence emission spectrum of the wild-type G6PD showed a peak at 338 nm (Fig. 5A). Three G6PD variants—G6PD Bangkok, G6PD Bangkok noi, and G6PD Songklanagarind—also showed an emission peak at around 338 nm. The other G6PD variants exhibited fluorescence emission shift to a longer wavelength (red shift). Furthermore, only G6PD Bangkok noi and G6PD Songklanagarind showed a fluorescence intensity lower than that of the native enzyme. The decrease or increase in fluorescence intensity observed in G6PD variants indicates the conformational changes. A reduction in fluorescence intensity observed in G6PD Bangkok noi and G6PD Songklanagarind might suggest that tryptophan residues were exposed to the solvent molecules. In the presence of NADP^+^, all recombinant enzymes showed reduced intrinsic fluorescence intensity (Fig. 5B). A decrease in fluorescence intensity and a red shift of G6PD variants implied that the tryptophan residues were exposed to an aqueous environment. This highlights the influence of mutations on the hydrophobic binding sites of proteins. Binding of G6PD to the NADP^+^ changes the conformation, causing an alteration in the local environment of the tryptophan residues.

ANS fluorescence analysis has been extensively used to monitor the conformational changes in proteins [33], [37]. ANS is an amphiphilic dye, the fluorescence intensity of which increases as it is exposed to a more hydrophobic environment. Consequently, binding of ANS to the protein is considered a suitable probe to monitor the hydrophobicity of protein surface. Many factors can attribute to alteration in protein surface hydrophobicity, including conformational change, protein denaturation, protein unfolding, oxidative modification and binding to some ligands. The blue shift (a shift to shorter wavelength) and an increase in fluorescence intensity generally indicates the increase in protein hydrophobicity. The ANS fluorescence spectra of the wild-type G6PD and the variants were monitored. Wild-type and G6PD Bangkok noi showed fluorescence spectra peak at 490 nm, whereas the other G6PD variants showed emission spectra with a shift to a shorter wavelength (blue shift; Fig. 5C). An alteration in ANS fluorescence spectra and an increase in fluorescence intensity of the variants (G6PD Bangkok, G6PD Songklanagarind, G6PD Union, G6PD Canton, G6PD Canton + Bangkok noi, and G6PD Union + Viangchan) indicated the conformational change and exposure to hydrophobic regions. Similar phenomena have also been observed in other G6PD variants such as G6PD Viangchan and G6PD Zacatecas [33]. It was reported that ANS can bind to cationic groups of the protein such as Arg and Lys. Ion paring is formed between the charged group of Lys or Arg and sulfonate group of ANS, decreasing the intermolecular charge transfer and leading to an increase in fluorescence intensity [39]. Interactions between Lys or Arg with ANS might induce a secondary structure formation and therefore has an effect on fluorescence intensity. As a consequence, the structural changes induced by ANS itself should be taken into account. However, binding of ANS should induce the structural change to the same extent for all G6PD variants as all of them contain the same number of Lys and Arg residues, except for G6PD Bangkok which has amino acid changed from Lys to Asn. This might be an explanation for similar fluorescence intensity to wild-type protein observed in G6PD Bangkok.

The conformational changes caused by mutations might partially contribute to the catalytic activity of G6PD variants. A small to moderate change in fluorescence intensity observed in G6PD Canton, G6PD Union and G6PD Songklanagarind resulted in a slight alteration in catalytic activity. A greater effect of structural change on catalytic activity has been demonstrated in G6PD Union + Viangchan. Both the intrinsic fluorescence and ANS fluorescence spectra of this variant were significantly different from those of the wild-type enzyme. This suggests that the combined mutation of Union and Viangchan greatly influences the local conformation of the G6PD Union + Viangchan variant. As a consequence, the catalytic activity of this double mutant was affected the most, reduction of approximately 2.5-fold in comparison to the native enzyme. It is worth mentioning that other factors such as alteration in secondary structure and defective in stability might also contribute to the catalytic activity and clinical phenotypes of G6PD variants.

### 3.3 Stability analysis of G6PD variants

**Fig. 6:**
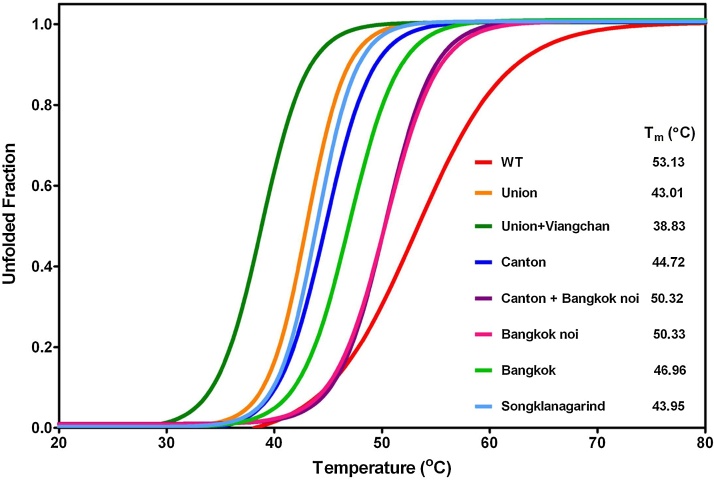
Thermal stability of recombinant human G6PD variants. Changes in the CD signal at 222 nm were monitored as the temperature increased from 20 °C to 80 °C. The melting temperature (*T*_m_) was defined as the temperature at which half of the secondary structure unfolded and was calculated for each G6PD variant.

To investigate further the effect of mutation on protein structural stability, the structural changes in G6PD variants were compared with the native enzyme by using CD analysis at 222 nm (Fig. 6). As temperature increased from 20 °C to 80 °C, the protein structure unfolded, and the melting temperature (*T*_m_) was defined as the temperature at which half of the secondary structure unfolded. The *T*_m_ of the wild-type enzyme ascertained in this study (53.13 °C) was close to those previously reported: 54.8 °C and 55 °C [25], [40]. The thermal denaturation of each G6PD variant was different and was dependent on the mutation site.

All of the seven clinical G6PD variants exhibited lower *T*_m_ than that of the native enzyme. Mutation at residue 501 in G6PD Bangkok noi slightly affected the structural stability of the protein—G6PD Bangkok noi and G6PD Canton + Bangkok noi showed a *T*_m_ value of around 3 °C lower than that of the wild-type enzyme. It should be noted here that G6PD Canton + Bangkok noi is thermally more stable than the single mutant, G6PD Canton. It is possible that the stability of the double mutant is likely to be more influenced by the G6PD Bangkok noi mutation. This also indicates the trade off between catalytic activity and stability in G6PD Canton + Bangkok noi—the activity is completely lost the enzyme must retain its stability. The global stability of the protein is altered in the presence of mutation at residue 275. G6PD Bangkok is less stable than the native protein, with a *T*_m_ value of 46.96 °C, which is 6 °C lower than that of the wild-type enzyme. In addition to disruption in salt bridge formation and alteration in secondary structure, the change in structural stability might also attribute to the complete loss of enzyme activity and CNSHA phenotype observed in G6PD Bangkok. Other G6PD variants were more sensitive to thermal denaturation, with G6PD Union, G6PD Union + Viangchan, G6PD Canton, and G6PD Songklanagarind showing respective *T*_m_ values of 43.01 °C, 38.83 °C, 44.72 °C, and 43.95 °C. G6PD Union + Viangchan was the least stable variant, showing a *T*_m_ value 13 °C lower than that of the native enzyme. This highlights the significant effect of Union and Viangchan mutations on the structural stability of the protein. Our results considering the thermal denaturation of G6PD Union + Viangchan are also in agreement with the intrinsic and ANS fluorescence analysis of the variant, confirming the impact of Union and Viangchan mutations on the global stability of the protein.

**Fig. 7:**
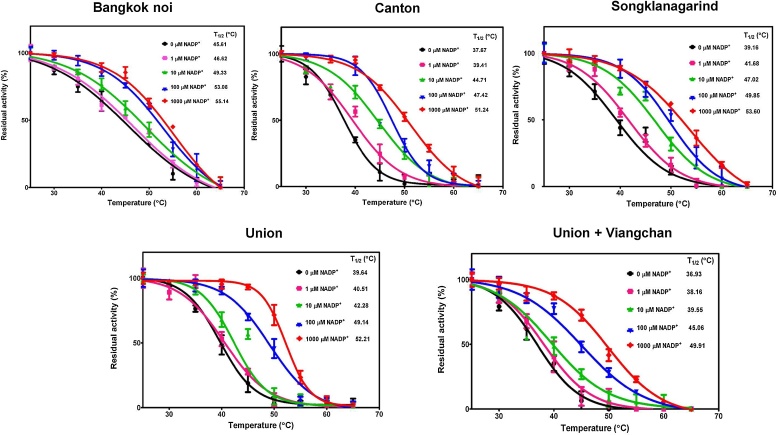
Thermal inactivation analysis of G6PD variants in the presence and absence of different concentrations of NADP^+^ (0, 1, 10, 100, and 1000 μM). *T*_1/2_ is the temperature at which the enzyme loses 50% of its activity.

The presence of a low concentration of NADP^+^ was found to increase the stability of G6PD [25], [30], [41], [42]. Each monomer subunit of G6PD contains one tightly bound structural NADP^+^ [42]. Removal of structural NADP^+^ leads to a loss of activity and causes considerable conformational alterations. The structural NADP^+^ prevents the dissociation of a dimeric enzyme into an inactive monomeric state [43]. Therefore, thermal inactivation of the G6PD variants was assessed in the presence and absence of different concentrations of NADP^+^—0, 1, 10, 100, and 1000 μM (Fig. 7). *T*_1/2_ was defined as the temperature at which the enzyme lost 50% of its activity.

In accordance with the previous reports, the presence of NADP^+^ improved the stability of the G6PD variants as the temperature increased in a manner that was dependent on the NADP^+^ concentration [25], [35]. In the absence of NADP^+^, the *T*_1/2_ values of G6PD Bangkok noi, G6PD Union, G6PD Union + Viangchan, G6PD Canton, and G6PD Songklanagarind were 45.61 °C, 39.64 °C, 36.93 °C, 37.67 °C, and 39.16 °C, respectively. This indicates that the G6PD variants are less stable considering that they showed a *T*_1/2_ lower than that of the wild-type enzyme (*T*_1/2_ = 47 °C) [25]. The presence of a physiological concentration of NADP^+^ (10 μM) was found to increase the *T*_1/2_ values of all variants, thereby considerably stabilizing G6PD Canton and G6PD Songklanagarind and increasing *T*_1/2_ for 8 °C from that of a condition where NADP^+^ was absent. The *T*_1/2_ values of all of the G6PD variants increased by 10 °C–15 °C in the presence of 1000 μM NADP^+^. It has been reported earlier that no NADP^+^-stabilization effect was observed for Class I G6PD variants such as G6PD Yucatan and G6PD Nashville [44]. Unfortunately, we were unable to investigate the thermal inactivation of the Class I variants (G6PD Bangkok and G6PD Canton + Bangkok noi) in this study because both variants showed no detectable enzymatic activity.

**Fig. 8:**
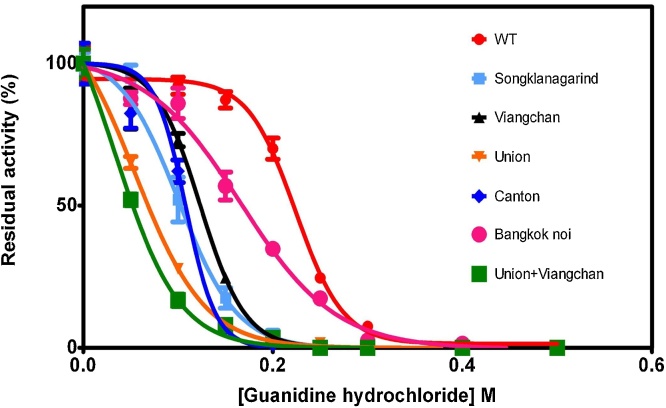
Stability analysis of G6PD variants in the presence and absence of varying concentrations of Gdn-HCl (0.05, 0.1, 0.15, 0.2, 0.25, 0.3, 0.4, and 0.5 M). The enzymatic activity was measured after incubation at 37 °C for 2 h. Residual activity of the enzyme was expressed as a percentage of the activity for the same enzyme incubated at 25 °C in the absence of Gdn-HCl.

Furthermore, the unfolding of G6PD variants was assessed in the presence and absence of different concentrations (0.05–0.5 M) of a chemical denaturant, guanidine hydrochloride (Gdn-HCl). Such an unfolding analysis using a denaturant is used to determine the conformational stability as the denaturant alters the tertiary structure of the protein [37]. When a protein is unfolded, its activity is perturbed; Fig. 8 shows the residual activity of the G6PD variants in the presence of an increasing concentration of Gdn-HCl after incubation at 37 °C for 2 h. G6PD Viangchan was also included in the study to evaluate the effect of Val291Met mutation on protein stability. As expected, wild-type G6PD was the most stable protein in the presence of Gdn-HCl, losing its entire activity in the presence of 0.4 M Gdn-HCl. All of the G6PD variants showed a lower structural stability than did the native enzyme. Although the native enzyme retained almost 100% of its original activity, the G6PD variants (apart from G6PD Bangkok noi) lost over half of their activity in the presence of 0.2 M Gdn-HCl. This is in agreement with the results of a previous report in which wild-type G6PD lost 50% of its original activity in the presence of 0.35 M [33]. Consistent with other structural analyses described above, G6PD Union + Viangchan was found to be the least stable enzyme, indicating that this variant is structurally unstable.

**Fig. 9:**
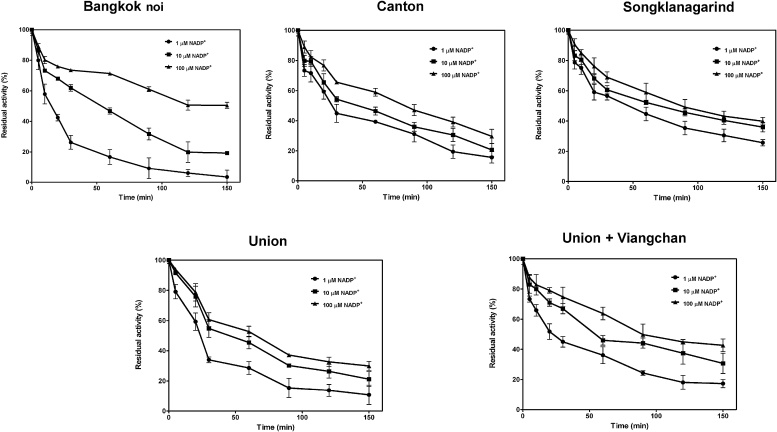
Susceptibility of G6PD variants to trypsin digestion in the presence of different concentrations of NADP^+^ (1, 10, and 100 μM).

Several studies have reported the effect of G6PD mutations on the susceptibility of the G6PD variants to protease degradation [25], [40], [45]. In this study, we investigated the susceptibility of five G6PD variants—G6PD Bangkok noi, G6PD Canton, G6PD Union, G6PD Songklanagarind, and G6PD Union + Viangchan—to trypsin digestion (Fig. 9). All of them were more susceptible to proteolysis when compared with the wild-type protein, as reported in the previous studies [25], [45]. In contrast to earlier studies, however, all five G6PD variants showed a significant increase in susceptibility to trypsin digestion. After 150 min of incubation and in the presence of 1 μM NADP^+^, all of the G6PD variants lost more than 80% of their original activity, except for G6PD Songklanagarind, which retained around 25% of its initial activity. G6PD Bangkok noi was the variant most susceptible to trypsin digestion in the presence of 1 and 10 μM NADP^+^ after incubation for 150 min. The accelerated degradation of the G6PD variants could be caused by defective protein folding, which also usually contributes to reduced protein stability as observed in G6PD-deficient individuals. In agreement with previous studies, the presence of NADP^+^ was found to counteract the susceptibility of the variants to trypsin digestion in a concentration-dependent manner [25], [40], [45]. The stabilization effect of NADP^+^ was markedly high for G6PD Bangkok noi; the presence of 100 μM NADP^+^ essentially increased the stability of this variant. This could be because the Bangkok noi mutation is located at the structural NADP^+^-binding site. However, it should be noted that the conditions of experimental *in vitro* digestion in this study are different from physiological settings where protein degradations occur in a proteasome, and the quality of protease is dissimilar.

It is important to note that mutation at residue 501 in G6PD Bangkok noi caused only a small to moderate effect on the secondary structure, thermal stability, protein conformational change and susceptibility to denaturant. However, G6PD Bangkok showed a significant reduction in catalytic activity of approximately 50-fold when compared to the native enzyme. Moreover, this variant is greatly susceptible to protease digestion. This might imply that G6PD Bangkok noi variant has a defect in protein folding. Substitution of Phe with Cys at residue 501 alters the size of structural NADP^+^ binding site and this might be causing the disruption in structural stability of the protein which could partially contribute to the defective in catalytic activity of G6PD Bangkok noi.

The results of CD analysis of protein secondary structure are in good agreement with other methods—intrinsic fluorescence and ANS binding assay, thermal stability, thermal inactivation analysis and susceptibility to denaturant and protease digestion for all G6PD variants except for G6PD Bangkok noi—it is more structurally stable than other variants but it is the most susceptible variant to protease degradation. G6PD variants with alteration in secondary structure of the protein are structurally unstable as they showed lower melting temperature (*T*_m_), lower *T*_1/2_ and higher susceptibility to denaturant and trypsin digestion than the native G6PD. The effect of mutation was most prominent in the double mutant, G6PD Union + Viangchan in which it is the least stable G6PD variant.

Considering both protein stability and catalytic activity, it was demonstrated that the overall fitness of G6PD variants i.e. good protein stability and activity, is a major contribution to the clinical phenotype of G6PD deficiency. The activity or stability alone does not determine or indicate the clinical outcome of G6PD variants. Indeed, a combination of both factors significantly attributes to severity of G6PD deficiency and, as a consequence, an essential determinant for separation of G6PD variants into different classes. The trade off between stability and activity observed in G6PD variants—G6PD Bangkok, G6PD Bangkok noi, G6PD Songklanagarind, G6PD Union, G6PD Canton, and G6PD Union + Viangchan determines the clinical features of G6PD variants. It is worth mentioning that G6PD activity is essential to retain the NADPH level and its stability is also critical in non-nucleated red blood cells, which contain no translational machinery. The coupling between protein stability and catalytic activity of G6PD variants was also described in another study [46].

## 4 Conclusions

In summary, the clinical manifestations observed in individuals with G6PD deficiency could be attributable to two major causes: the reduction in the catalytic activity of the G6PD variants or the decrease in the number of active G6PD molecules. To our knowledge, the present study is the first to report that Bangkok (Lys275Asn) and a combination of Canton + Bangkok noi (Arg459Leu + Phe501Cys) mutations dramatically affect the catalytic efficiency of the G6PD variants, as both G6PD Bangkok and G6PD Canton + Bangkok noi variants completely lose their enzymatic activity. There was a 50-fold reduction in catalytic activity of G6PD Bangkok noi compared with the catalytic activity of native enzyme. This indicates that replacement of Phe501 alone makes a significant contribution to the loss of catalytic activity observed in the double mutant. Mutation at Lys 275 disrupts the salt bridge formation, whereas the Phe501Cys mutation alters the size of the NADP^+^-binding site, thereby diminishing the catalytic activity of the protein. However, these mutations showed only a moderate effect on protein stability. Other mutations such as Songklanagarind (Phe66Ile), Union (Arg454Cys), and Canton (Arg459Leu) have an effect on the catalytic activity and stability of G6PD to different extents. G6PD Union, G6PD Canton, and G6PD Union + Viangchan showed a small change in catalytic efficiency of G6PD. These mutants present an approximately two-fold decrease in the catalytic activity when compared with the wild-type enzyme. G6PD Songklanagarind displayed a catalytic efficiency comparable to the wild-type G6PD. The presence of Songklanagarind, Union, Canton, and Viangchan mutations significantly affects the stability of the G6PD variants. It is notable that the combination of Union and Viangchan mutations considerably reduces the stability of G6PD Union + Viangchan variant. Considering the Class II variants, our results suggest that protein instability in non-nucleated erythrocytes is the principal factor contributing to the clinical manifestations. In contrast, kinetically defective enzymes cause severe hemolysis as seen in the case of Class I variants. Altogether, our present study provides valuable information regarding the molecular mechanism underlying the clinical features observed in individuals with G6PD deficiency. Moreover, the results demonstrate that the clinical manifestations are mainly determined by the trade off between protein stability and catalytic activity. The summary of a “trade off” for each variant is demonstrated in Table 3.

**Table 3:** A trade off between catalytic activity and stability of recombinant human G6PD enzymes.

Construct	Class	Catalytic activity (*k_cat_)*^a^	Stability^a^	Secondary structure^a^	Clinical phenotype
Wild-type	–	Normal	Normal	Normal	Normal
Bangkok	I	Complete loss	Moderately decreased	Changed	Severe deficiency with CNSHA
Bangkok noi	I	48-fold decreased	Slightly decreased	Changed	Severe deficiency with CNSHA
Songklanagarind	II	Similar to normal enzyme	Markedly decreased	Changed	Severe deficiency
Union	II	2-fold decreased	Markedly decreased	Changed	Severe deficiency
Canton	II	2-fold decreased	Markedly decreased	Changed	Severe deficiency
Union + Viangchan	II/III	2-fold decreased	Markedly decreased	Changed	Severe deficiency
Canton + Bangkok noi	I	Complete loss	Slightly decreased	Changed	Severe deficiency with CNSHA

a When compared with wild-type enzyme.

## Author contributions

Conceived and designed the experiments: UB. Performed the experiments: UB, KC, TS, and TJ. Contributed reagents/materials/analysis tools: UB, ND, NW, and MI. Wrote the manuscript: UB, and MI.

## Conflicts of interest

The authors declare that there is no conflict of interest.
